# Efficacy of Bacteriophages in Wound Healing: An Updated Review

**DOI:** 10.7759/cureus.71542

**Published:** 2024-10-15

**Authors:** MP Narayanan, Ankur Kumar, Ganesh Kumar Verma, Avinash Bairwa, Anissa A Mirza, Bela Goyal

**Affiliations:** 1 Biochemistry, All India Institute of Medical Sciences, Rishikesh, Rishikesh, IND; 2 Microbiology, All India Institute of Medical Sciences, Rishikesh, Rishikesh, IND

**Keywords:** antibiotic resistance, bacteriophages, chronic wounds, infection control, wound healing

## Abstract

We have attempted to summarize the efficacy of bacteriophage therapy, highlighting the recent advances and phage delivery methods in different clinical trials and animal studies for wound-associated infections. Bacteriophage therapy is the lyse of bacteria by bacteriophages at the site of invasion. As bacteria become more resistant to antibiotics, discovering an alternative is more important than ever, and bacteriophage therapy has yielded promising outcomes. A clear knowledge of the bacteriophage, microbiota, and human host and their interaction is necessary to implement bacteriophage treatment on a large scale. Much technological advancement and regulatory guidelines increased the credibility of phage therapy (PT). Still, the challenges include the development of efficient bacteriophage screening methods, phage therapy strategies for biofilms, and the quality and safety of phage preparations. However, much consideration is to be taken in designing a novel therapeutic approach for antibiotic-resistant infections by using phages, phage lytic proteins, bioengineered phages, or antibiotics in combination.

## Introduction and background

Antibiotics are the most effective therapeutic agents, as they saved millions of lives in the history of modern medicine. However, as time progresses, antibiotic resistance reduces its effectiveness, leading to the rise of multidrug-resistant (MDR) bacteria as the primary cause of havoc today. The World Health Organization (WHO) indicated that the annual deaths due to antibiotic resistance could reach ten million in 2050 [1]. Chronic non-healing wounds affect about 605 million patients with financial burden, isolated life, or depression, causing significant morbidity and mortality globally [2]. This threat paved the way for discovering other therapeutic agents, including phages. Phage therapy (PT) is also known as living medicine because living organisms are used for treatment. Bacteriophages are a type of virus that can infect and lyse bacterial cells. Structurally, it contains protein and nucleic acids. They attach to the receptors of bacteria and transfer the genome into them. Then phage replication in the bacterial cytoplasm is carried out, and new lytic bacteriophages are released. The process is repeated and results in a decrease in specific bacterial populations. Phages are found where bacteria thrive in sewers and rivers like Ganga [3]. Hankin found the antibacterial characteristics of Ganga and Yamuna water, and even raw water from these rivers can cure diarrhoea and cholera [4]. Later, it was shown that some heat-labile antibacterial properties that killed *Escherichia coli* were present in Ganga water [5]. Besides the antibacterial properties, phages protect humans, including autoimmune diseases and allergies, by restoring immunological homeostasis [6]. Several studies have been conducted regarding Ganga water's healing properties, including phage isolation and their therapeutic uses [7].

**Figure 1:**
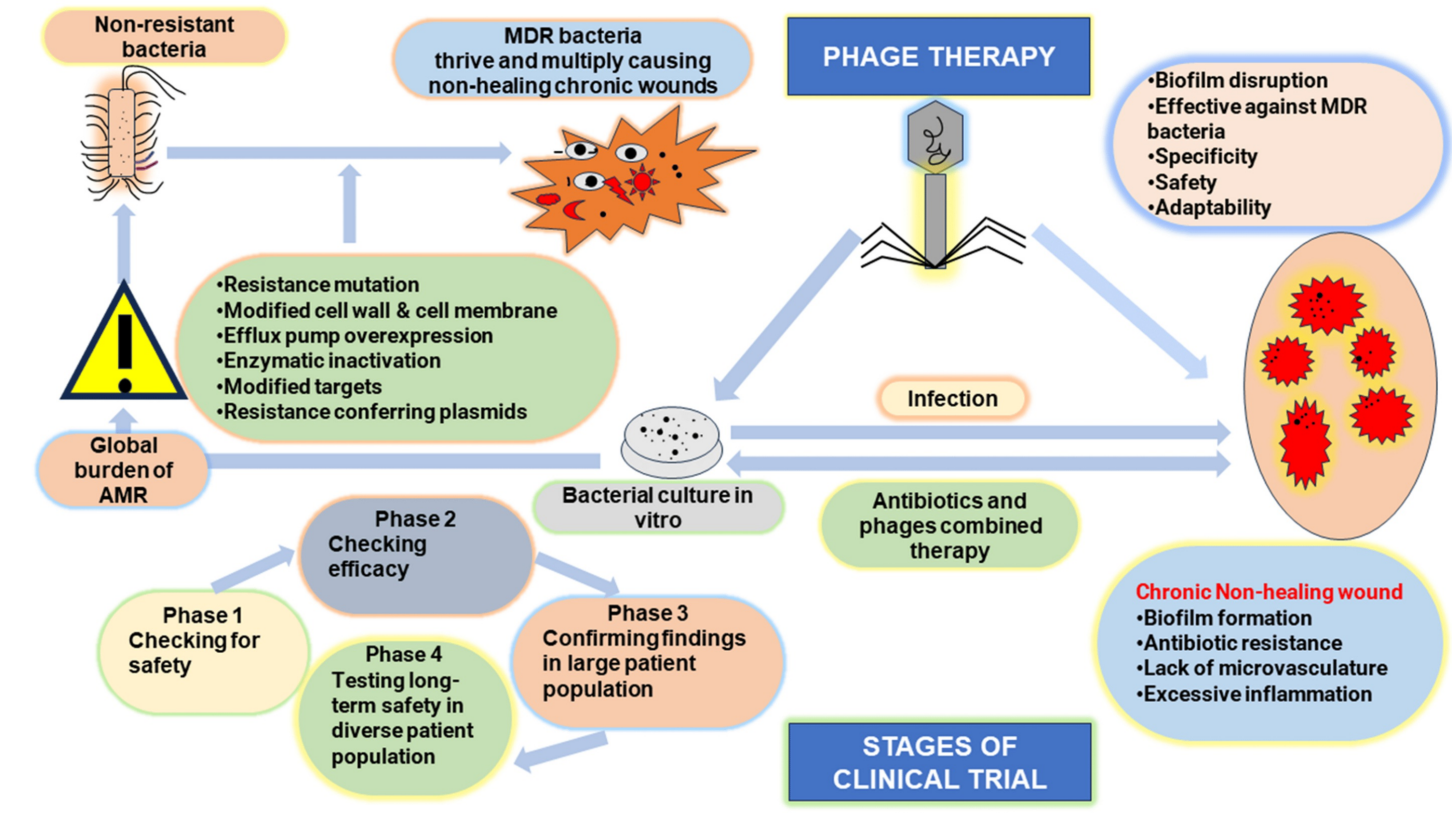
Depiction of phage therapy and clinical trial Different causes of AMR, global burden of AMR, challenges in non-healing wound treatment, antibiotic and phage synergy, advantages of PT and different phases of clinical trial. Image credit: Author's own creation.

PT is only effective if the strain matches the disease-causing organism. Hence, phage mixtures or cocktails are used to increase the rate of successful PT [8]. It is found that phages taken from disease-recovering patients are very effective in treating the same MDR strain-specific bacterial infections [9]. New phages are added to the phage cocktail to fight against the emerging antibiotic-resistant pathogenic bacterial strains [10]. PT is successfully used in polysaccharide layer-covered biofilms from MDR bacterial infections where antibiotics fail to penetrate [11]. Some advantages of PT are specificity, universality, self-dosing and self-limiting ability, non-toxicity, anti-biofilm activity, evolution, simple production, and formulation. In 2015, personalized PT was successfully used against *Acinetobacter baumannii* [12]. The first Food and Drug Administration (FDA)-approved PT clinical trial was designed in 2019 in the United States of America (USA) [13]. Then, a successful clinical trial against *Pseudomonas aeruginosa* infections was conducted at Yale University in 2020 (NCT04684641) [14]. Figure 1 illustrates PT, including antimicrobial resistance (AMR), MDR bacteria, phage antibiotic synergy, and clinical trials. In this review, we examine the current status of PT, including its biological underpinnings, therapeutic uses, unmet obstacles, and potential future paths.

## Review

Different types of wounds

Chronic wounds are primarily a public health issue brought on by age, high diabetes prevalence, and obesity. Depending on the time taken for wound healing, wounds are classified into (a) chronic infection wounds, (b) difficult-to-heal wounds, and (c) nonhealing wounds [15]. Approximately 5 to 7 million patients are affected by chronic infection wounds each year. In the USA, skin-related infection alone treatment costs about 75 billion USD [16]. PT can be effectively used for a variety of wound infections that are difficult to manage by conventional antibiotics, including recurrent urinary tract infection (UTI), chronic rhinosinusitis and otitis media, skin and soft-tissue bacterial infections, respiratory tract infections, prosthetic joint infections, infections related to cardiac devices, endovascular infections, traumatic wound infections, surgical wound infections, burn wound infection and diabetic wound infections. The location of wound-associated infection is crucial for antibiotic therapy. The antibiotic has to cross the blood-brain barrier to achieve the minimum inhibitory concentration in bacterial meningitis. Chronic leg and foot ulcers are challenges to healthcare systems. They are classified as venous wounds, arterial wounds, diabetic foot ulcers (DFU), and pressure wounds. The major causes of chronic wounds are metabolic disturbances (e.g., diabetes mellitus), vascular supply deficits (e.g., venous and arterial insufficiency), and mechanical impacts like localized pressure. PT can be used against MDR *P. aeruginosa* biofilm to increase the permeability of antibodies to the biofilm. Phages produce bactericidal enzymes like depolymerase, lysozymes and other lytic enzymes to destroy biofilm. Combined use of bacteriophages, antibodies, nanoparticles, enzymes, and natural products will more effectively invade and destroy the biofilm [17-19].

Mechanism of PT in wound healing process

Bacteriophages provide a selective and highly focused approach for treating MDR bacterial infections. The effectiveness of PT in wound healing involves major mechanisms like bacterial lysis, disruption of the MDR bacterial biofilm, immune response modulations, and combined use, i.e., synergy with antibiotics. These mechanisms are crucial to facilitating non-healing wound therapy by overcoming the barriers of traditional antibiotic therapy.

Bacterial Lysis

The lytic cycle is an important mechanism of PT. In the lytic cycle, phages attach to the bacterial cell, then their genetic material is injected into the bacterial cell, and new phage particles are synthesized using bacterial machinery. This process results in bacterial cell death and the release of new phages that can infect the nearby bacterial cell. This targeted bacterial destruction is advantageous in non-healing chronic wound infections where the MDR bacteria hinder natural healing. Penetration of the phage tail tubes and the injection of viral particles into the bacterial cell happen with the help of phage lytic enzymes. Virion-associated lysins stimulate the breakdown of cell wall peptidoglycans, which causes the breakdown of the infected cell and the release of new viral particles. Few endolysins are exported by bacterial cell machinery, while the majority enter the cell wall through holin channels.

Specificity of Bacteriophages

Bacteriophages have the powerful advantage that they are highly specific in targeting only their host bacteria without affecting the normal microbiome. The application of phages as therapeutics - a viable substitute for antibiotics - heavily depends on identifying lytic phages that are efficient against the target bacteria. The selection methods used now are time-consuming and do not enable immediate visualization of the dynamics of phage infection. Egido et al. describe a reliable way of selecting phages by real-time monitoring of phage infection using fluorescent DNA dye. This method allows high throughput scaling and real-time fluorescence signal readout monitoring of the phage infection. This is accomplished by staining the DNA of lysed bacteria and new phages with a membrane-impermeant nucleic acid dye [20].

Biofilm Disruption

Bacterial populations that are organized and encased in their extracellular matrix are known as biofilms. Biofilm structure protects the bacteria from the immunological actions of the host and the antibiotics. This contributes to the persistence and chronic nature of the non-healing wound. Bacteriophages are excellent at penetrating these biofilms and enhancing their therapeutic effectiveness [21]. These phages are specifically selected or engineered to disrupt the protective biofilm. They can lyse the bacteria within the biofilm and can improve wound healing. The biofilm components are polysaccharides, proteins, extracellular DNA, and lipids. Phages produce enzymes (e.g., depolymerase) that break down biofilm matrix components. Some notable pathogens, like *P. aeruginosa* and *Staphylococcus aureus*, are common culprits in chronic nonhealing wounds. Other biofilm-forming bacteria and phages used against them are detailed in Table 1.

**Table 1:** Phage therapy used for destruction of biofilm-forming MDR bacteria XDR: extensively drug-resistant; MDR: multidrug-resistant.

Biofilm-forming bacteria	Bacteriophage	Study references
*Acinetobacter baumannii *extensively drug-resistant (XDR)	Bacteriophage AB1801	[22]
*A. baumannii *(MDR)	Phage lysin PlyF307	[23]
*P. aeruginosa* 1193	Lytic IME180 bacteriophage depolymerase enzyme	[24]
(PDR) *Klebsiella pneumonia* UA168	Phage KP168	[25]
*K. pneumonia* (MDR)	Depolymerase enzyme encoded by phage SH-KP152226	[26]
*P. aeruginosa* PAO1	Bacteriophage vB_PaeM_SCUT-S1 Bacteriophage vB_PaeM_SCUT-S2	[27]
Methicillin-resistant *S. aureus *(MRSA)	Phages UPMK_1 and UPMK_2	[28]
P. aeruginosa (MRSA, MSSA)	Bacteriophage CSA13	[29]
*P. aeruginosa* PA14	Phage JBD4 and phage JBD44a	[30]
*P. aeruginosa* PAO1 and ATCC 10,145	Bacteriophage PhiIBB-PAA2 and Bacteriophage phiIBB-PAP21)	[31]

Enzymatic Degradation

Penetration and deep spread of phages into the biofilm is facilitated by phage-produced enzymes like depolymerase, which helps in degrading the biofilm matrix. The biofilm disruption makes the resistant bacterial cell accessible to the phages as well as the host immune system. Then, the phage-encoded enzymes degrade the biofilm matrix constituents like nucleic acids, proteins, and polysaccharides and promote the weakening of the biofilm structure, causing bacterial cell lysis [32].

Modulation of the Immune Response

The mechanisms of interactions between phages, bacteria, and the host immune system are very complex and multifaceted. Phages can influence the host immune system and boost its effect on wound healing. Phages can modulate the host immune response by immunomodulation, possibly lowering high levels of inflammation that may prevent wounds from healing. Based on the available data, the effect on the host immune system is phage-specific. It depends on the antibacterial activity of phages. By reducing bacterial load and inflammation, phages create an environment for tissue repair and regeneration [33].

Phage-Antibiotic Combination Therapy

Phage antibiotic synergy works through different mechanisms like increasing antibiotic susceptibility and depolymerization of the bacterial polysaccharides by phage enzymes that promote antibody diffusion and cell penetration. This will lead to a reduced dosage of antibiotics, minimal side effects, and a reduced risk of resistant development. It was shown that phages are effective in reversing the bacterial resistance in some strains. This can expand the effectiveness of weaponry against MDR bacteria [34]. The major mechanisms of action of bacteriophages in wound healing through phages and antibiotic synergy, modulations of the immune response, biofilm disruption, and bacterial lysis emphasize their potential as powerful agents in treating MDR bacterial infections.

By all these mechanisms, PT offers an effectively targeted and multifaceted therapeutic approach towards chronic wound infections, overcoming the limitations of antibiotic treatment and opening a new way for improved patient outcomes and reducing healthcare costs in wound treatment. In the literature, there are various approaches discussed to promote healing and protect from complications in chronic wounds. These include antibiotic and antimicrobial dressings, advanced dressings, negative pressure wound therapy, adjuvant therapies, and pain management. PT is more effective and successful in treating MDR bacterial infections among these methods. With minimal side effects, it protects healthy microbiota and breaks biofilms. They are safe and sustainable and can be used as an adjunct therapy with antibiotics [35-36].

Consideration and preparations for PT

**Figure 2:**
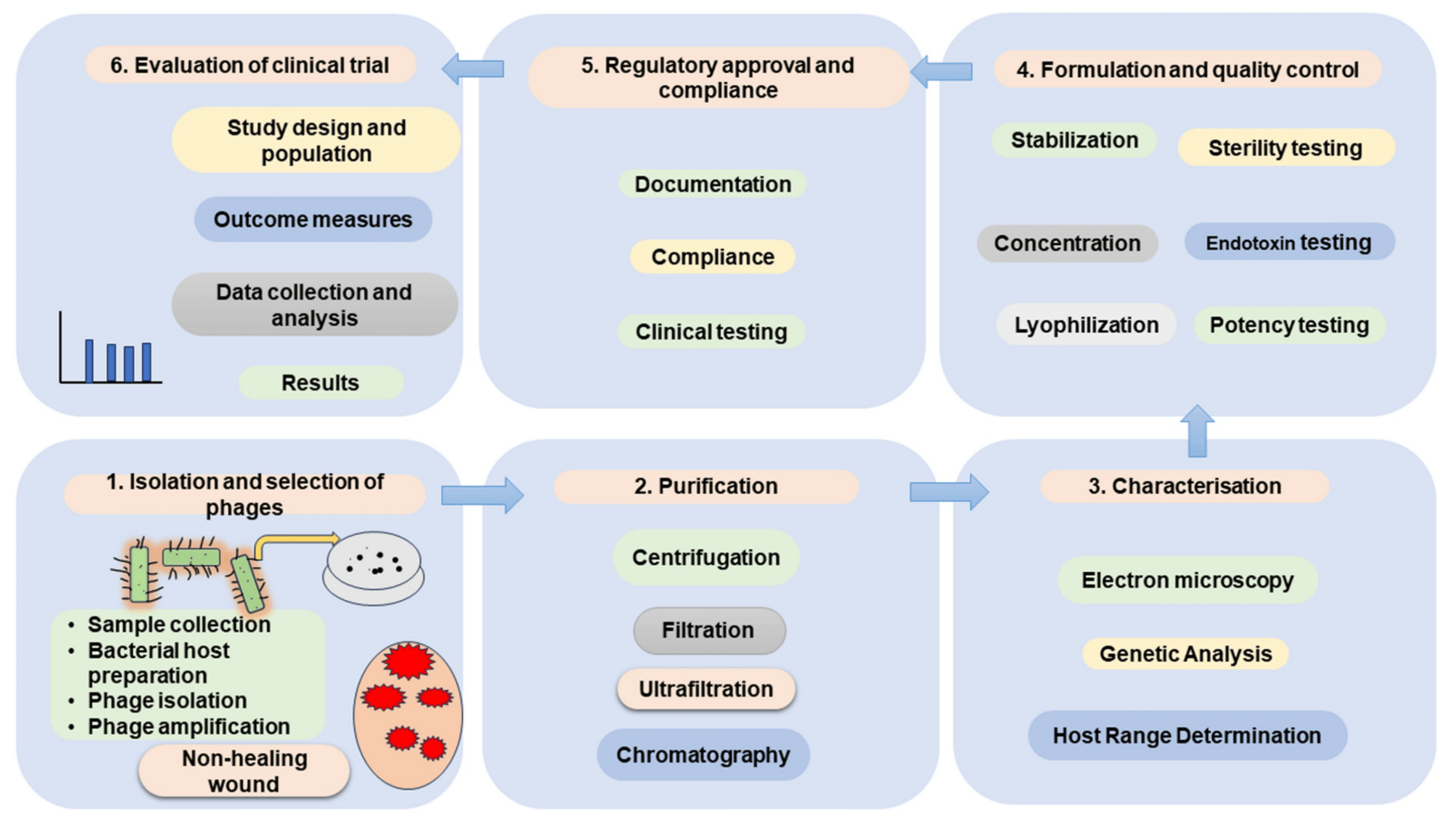
Steps in preparation of phage suspension Steps in preparation of phage suspension, screening, purifying, characterization, formulating, regulatory approval and evaluation of clinical trial. Image credit: Author's own creation.

Identification and selection of phages is the first step. Apart from finding out for new phages, it is also possible to use those that are available in the phage bank. If the host bacterium is culturable, isolation of phages is easy. Pure phage lysate can be obtained by centrifugation, membrane filtration, and other purification methods. Bioreactors can be used for large-scale methods. Centrifugation and ultrafiltration are different steps to avoid the presence of bacterial toxins, lipopolysaccharides, and other cellular debris in phage suspension. Phages can be stored at -80 °C or freeze-dried. Figure 2 summarizes the primary procedures for producing phage suspensions appropriate for use in clinical settings.

Phage preparations are susceptible to protease action, high temperature, pH variations, and ionic strength [37]. So, it is important to use an appropriate formulation. Delivery strategy is based on the site of infection and requires careful consideration. Table 2 lists different phage preparations.

**Table 2:** Different bacteriophage carriers/preparations used for therapy

Phage carriers/preparations	Mechanism of action
Polymeric carriers	Enhances acid resistance and phage titer in the gastrointestinal tract
Transdermal delivery by microneedle	Exert systemic therapeutic effects by penetrating the skin
Electrospun fibers carriers	Controlled release of phage by the selection of polymer materials used
Phage‒antibiotic synergy (PAS)	Combination therapy results in maximum bacterial killing
Phage creams/ointments	Non-ionic-based creams for topical application of phages
Phage cocktail	Even resistance occurs the non-resistant phage continue to work
Hydrogel fibers	Phage-enclosed hydrogel fibers slowly release lytic phages at the site of infection
Liposome-encapsulated bacteriophages	Cationic liposome loaded phages increase therapeutic efficiency

Recent advances in PT for wound healing

Several technological and methodological advancements in the field have propelled PT into the forefront of all wound care research. Enhanced phage delivery systems, genetically engineered phages, synthetic phages, and phage cocktail development are recent advancements in PT for wound healing. These make PT a promising alternative to traditional antibiotics. Enhanced phage delivery systems include hydrogels, nanoparticles, bandages, and dressings, which allow sustained phage delivery at the infection site. Genetically modified phages have enhanced lytic capabilities and broader host ranges. Through genetic modifications, phages can express specific adhesion proteins. Such genetically engineered phages are capable of affecting a broader range of bacterial species. The CRISPR-Cas system allows gene editing in phages to enhance their specificity and biofilm lytic activity [38]. Synthetic phages are tailored to specific bacteria, increasing the precision and effectiveness of therapy. A phage cocktail with multiple phages for different bacterial strains increases robustness and effectiveness in treating complex non-healing wounds [39]. Advanced phage delivery systems include hydrogels, liposomes, and nanoparticles [40].

Clinical applications

Preclinical Studies

Understanding the safety, efficacy, and mechanism of action of bacteriophages in wound healing requires preclinical animal study. By comprehending how phages engage with both host tissue and the immune system, scientists may foresee possible challenges and enhance therapeutic protocols. This step is critical for the scientific proof to go to clinical trials. Oral phage treatment prevented 66.7% of mice subjected to *P. aeruginosa*-induced gut-derived sepsis from dying [41]. Experimental PT in a mouse model against *P. aeruginosa*, *Yersinia pestis*, and *Burkholderia cepacia* has been successful, suggesting that phages are promising alternatives against antibiotics [42]. PT as a single dose administered concurrently with *Clostridium difficile* administration was found to be sufficient prophylactic against infection in a hamster model of *C. difficile*-induced ileocolitis; phage treatments administered after infection preserved 11 out of 12 mice, whereas control animals given *C. difficile* and clindamycin died in 96 hours [43]. Using a hamster model, phage combinations also dramatically suppressed *C. difficile* growth in vitro and proliferation in vivo [44]. In bacteremia studies using vancomycin-resistant *Enterococcus faecium* [45], imipenem-resistant P. aeruginosa [46], and extended-spectrum β-lactamase-producing *E. coli* [47], a single phage strain given intraperitoneally was sufficient to save all the animals. Phage mixtures have also been used in animal models to treat respiratory tract and gastrointestinal tract infections caused by *P. aeruginosa* that are resistant to antibiotics [48]. Other animal studies, including MDR *E. coli* O25:H4-ST131 [49], Vibrio parahaemolyticus [50], *S. aureus* [51], and *A. baumanii* [52], had similarly positive results. The example of MDR *P. aeruginosa* shows how phage-resistant bacteria can restore their sensitivity to antibiotics [53].

Rezk et al., in a study with ZCPA1 phage, demonstrated complete wound healing in animal models and were highly specific against MDR *P. aeruginosa*. Hospital-acquired wound infection caused by *P. aeruginosa* has a high mortality rate and is prevalent in countries like Egypt [54]. In both clinical trials and animal models, numerous studies demonstrated the effectiveness of PT for *P. aeruginosa*-caused cystic fibrosis. Seth et al. used an animal study to examine the efficacy of PT in treating *S. aureus* biofilm wounds, and the combination cocktail PT was evaluated as successful [55]. MR-5 and MR-10 phage cocktails were used in a study conducted at Punjab University, India. In this animal study, liposome formulation was successfully experimented with in diabetic wounds [56]. In another clinical study by the same authors reveals the effectiveness of the phage cocktail MR-10 in treating diabetic wounds. Kishore et al., in their animal study, used MRSA-specific phages to treat osteomyelitis successfully [57]. Patel et al. used a phage cocktail to treat MDR and XDR Acinetobacter baumannii strains in animal models. They concluded that prior clinical studies are required for patient therapy [58].

Clinical Studies

Nowadays, the effectiveness of PT is represented by several successful case reports across the world. However, research, clinical trials, and standardization are necessary for the transition of these therapeutic agents into common-place adjuncts to antibiotics. Clinical trials are essential to assess the efficiency or side effects and to establish dosage recommendations. In 2002, scientists prepared a biodegradable polymer substance known as PhagoBioDerm, which was impregnated with phages and antibiotics. Ulcers and wounds containing *P. aeruginosa*, *E. coli*, *S. aureus*, *Streptococcus*, and *Proteus* were successfully treated in 70% of patients using this PhagoBioDerm [59]. The major bacteria responsible for infections and colonization in nonhealing wounds or venous leg ulcers are *S. aureus*, *P. aeruginosa*, *E. coli*, *E. faecalis*, coagulase-negative staphylococci and Proteus spp., which are listed as priority pathogens for the development of new antibiotics by the WHO [60]. Clinical trials need to be efficacious against such priority pathogens, and for that, the right phage in the right dose must be delivered to the infection site with enough susceptible bacteria [61]. PT has different advantages compared to antibiotics, like cost-effectiveness and fever side effects, and they destroy the bacterial load without affecting the intestinal flora. While antibiotic use leads to drug resistance, phages destroy bacterial viability forever. Their reproductive capacity increases the effectiveness of bactericidal activity. Self-renewing phages, low stability, release of endotoxins, and phage-insensitive mutants are some challenges to PT [62].

Data obtained from http://www.Clinicaltrials.gov shows 16 completed and 21 recruiting bacteriophage clinical trials. The safety and tolerability of TP-102, a bacteriophage cocktail, as a topical treatment for diabetic foot infections caused by *S. aureus* and *P. aeruginosa* are assessed in the DFU Clinical Trial (NCT04803708). The MUCOPHAGES study (clinical trial NCT01818206) demonstrated that bacteriophages infect their bacterial hosts in the sputum regardless of the clinical characteristics of the patients, which was a significant step towards the development of bacteriophage treatment for persistent lung-associated infections. Table 3 lists recent clinical trial results.

**Table 3:** Recent clinical trials using phage therapy

Clinical trial number	No. of participants	Study design	Type of infection	Bacteriophage therapy	Status/outcome
NCT04803708	20	Randomized, double-blinded study	DFU (*P. aeruginosa* infection, *S. aureus* infection, *Acinetobacter* infection)	Bacteriophage cocktail (TP-102)	Completed assessed the efficacy in reducing bacterial load and promoting wound healing. The trial demonstrated that TP-102 was safe and well-tolerated, with promising signs of efficacy in reducing bacterial infections and enhancing wound healing in DFU
NCT01818206	60	Intervention model	Cystic fibrosis infection (*P. aeruginosa*)	Ten bacteriophages	Published. Data indicate that PT is generally well tolerated and can provide favourable clinical outcomes in a sizable population of patients with chronic bacterial infections that would otherwise be incurable.
NCT03140085	113	Randomized, double-blinded study	Urinary tract infection (UTI)	PYO bacteriophage therapy	Successful in treating UTI, safety level was good.
NCT05498363	100	A retrospective, observational analysis	Chronic bacterial infections	Individual bacteriophages and six bacteriophage cocktails	PT was successful in difficult-to-treat infections and it can be effective in combination with antibiotics
NCT04684641	8	Randomized, placebo-controlled, double-blind study	Cystic fibrosis (*P. aeruginosa*)	Phage YPT-01	Successful therapy for reducing sputum bacterial number
NCT04596319	29	Double-blind, randomized, placebo-controlled study	Lung infections cystic fibrosis (*P. aeruginosa*)	Phage cocktail AP-PA02	Completed results highlight the potential of PT as a novel approach to treating chronic bacterial infections, especially those resistant to antibiotics, in CF patients.
NCT04682964	128	Interventional	Tonsillitis	Nebulized phage cocktail	Estimated date of completion December 2024
NCT03808103	30	A phase 1/2a double-blind, randomized, placebo-controlled trial	Crohn's disease	Phage preparation EcoActive	Estimated date of completion September 2024

The two most well-known Eastern European institutes where human PT clinical trials have been conducted for nearly a century are the Eliava Institute and the Institute of Immunology and Experimental Therapy in Poland. Phage has been widely employed by the Eliava Institute to treat common bacterial pathogens in both preclinical and clinical settings, including Salmonella spp., *E. coli*, *S. aureus*, *Streptococcus spp.*, *P. aeruginosa*, *Proteus spp.*, *S. dysenteriae*, and *Enterococcus spp.* [63]. Topical treatment of phage specific to *S. aureus* was all that was required for each of the six patients with DFU that were not healing with antibiotics [64]. A phage cocktail composed of several phages targeting *Shigella flexneri*, *Shigella shiga*, *E. coli*, *Proteus spp.*, *P. aeruginosa*, *Salmonella typhi*, *Salmonella paratyphi* A and B, *Staphylococcus spp.*, *Streptococcus spp.*, and *Enterococcus spp.* was the only treatment administered to 219 patients with bacterial dysentery in a 1938 clinical trial. Oral and rectal administration of cocktails was used. In just two to three days, 27% of patients with blood in their stools reported improvement, and 28% reported alleviation from this symptom within 24 hours. Of the 219 individuals, 74% had their symptoms resolved or improved [65].

A clinical trial was conducted by Wright et al. in otitis patients with Biophage-PA. All 24 patients with a disease history of even fifty years are successfully treated by PT. Such phages also proved effective in burn wounds, lung infections, and cystic fibrosis. Thirteen clinical trials on PT carried out between 2005 and 2021 demonstrated the treatment's safety and lack of side effects. Six of these trials were unable to demonstrate the effectiveness of PT. However, two investigations by Ooi et al. and Wright et al. proved the efficacy of the phage cocktail they employed in their clinical trials. The other five studies also failed to demonstrate PT's efficacy but addressed the technical aspects and challenges during the study [66-67].

In a study from India, 21 patients with chronic non-healing wounds and 27 patients with DFU were treated by personalized PT. After three months of PT, complete wound healing was achieved. Diabetic foot infections are the primary cause of amputation in 20% of severe diabetic patients. PT has also been effective in treating chronic wounds such as persistent venous ulcers [68]. Suh et al., in their review of clinical trials, reported the successful treatment by PT for recurrent UTIs, sinusitis, nonhealing chronic infections of the skin, sepsis, and bone-related inflammations [69]. Międzybrodzki et al. conducted a clinical study on 153 patients suffering from chronic non-healing wounds. The patient’s efficacy and safety analysis were satisfactory, and the bacterial-resistant infections were healed completely after PT [70]. Rose et al. conducted phage BFC-1 therapy in nine patients with burn wounds. No adverse effects were reported in the patients who underwent the therapy [71]. Kvachadze et al. reported a case study in which Sb-1 phage was used against MDR bacteria in cystic fibrosis. The PT concludes with satisfactory results, and no negative effects were reported [72]. According to Nadareishvili et al., four surgical infection patients with osteomyelitis, DFU, and skin transplant surgery received PT, and the lesions of all four patients healed after a month of PT without any negative side effects [73]. Eliava Institute in Georgia produced a phage cocktail targeting common bacteria in wound infections. Such types of phage cocktails are manufactured in Georgia, Russia, and Poland [74]. Phagoburn was a European clinical trial to treat wounds due to burns, but this PT in 26 patients for 17 months was less effective, and patients had fewer adverse effects [75].

In a clinical study, 39 patients were included with diabetic wounds showed a promising treatment against antibiotic-resistant bacteria [76]. *S. aureus*-infected diabetic ulcers were treated in nine patients with combined PT and standard wound care. MDR bacteria in diabetic wounds are also treated with Pyo bacteriophage [77]. *S. aureus* and *P. aeruginosa* infections present in burn patients are treated with phage spray. In this study, patients showed fewer abnormalities, and the number of MDR bacteria was significantly decreased [78]. Gupta et al. treated twenty patients with antibiotic-resistant wounds with a phage cocktail for different doses, which led to successful healing [79]. In another study, an AB-SA01 phage composed of myoviruses was designed that was specific against *S. aureus* bacteria. Its efficacy was evaluated and found safe [80]. McCallin et al. conducted a genomic analysis of different cocktails of bacteriophages used for *S. aureus* in chronic skin and wound infection patients [81]. Kifelew et al. tested the efficacy of the AB-SA01 phage cocktail for treating MDR-*S. aureus* infections of DFU. The therapy was completed without adverse effects, and a decrease in bacterial load resulted in early wound healing [82]. In another study conducted by Wang et al., lysin from Staphylococcus phage JD007 and a cell-penetrating peptide were used in combination to kill MRSA skin infections [83]. U.S. National Library of Medicine, http://www.Clinicaltrials.gov, showing ongoing or completed bacteriophage clinical trials, which involve eight in bacterial infections and UTI, five in prosthetic joint/joint infections, five in chronic wounds, four each in cystic fibrosis and DFU, one each in acute tonsillitis, diarrhea, and necrotizing enterocolitis.

From these clinical trials and animal studies, it is clear that phages can be used in chronic antibiotic-refractory infections, purulent infections, wound prophylaxis, poorly accessible infections, chronic otitis, respiratory tract infections, UTIs, sepsis, etc. Non-healing wounds are health burdens that require prolonged hospitalization due to the presence of drug-resistant bacteria. Phages are natural weapons with bactericidal properties that several clinical studies have proved. Proper regulatory rules must be followed for the authorized and legal use of bacteriophages for patient therapy. In this regard, all clinical trials should be controlled by a legal framework for medical application and compassionate therapy, as mentioned by the Helsinki Declaration [84]. PT for different nonhealing wounds/diseases and phages used are listed in Table 4.

**Table 4:** Phage therapy for different diseases/wounds

Diseases/wounds	Pathogen	Phage	Outcome
Skin graft infections	P. aeruginosa	Phage BS24 [85]	This study demonstrated the effectiveness of PT in preventing the destruction of skin grafts caused by *Pseudomonas aeruginosa* infections.
DFU	S. aureus, P. aeruginosa, Acinetobacter baumannii	Anti-Staphylococcus bacteriophage therapy Liquid/liposome phage cocktail phage cocktail AB-SA01 [86]	The bacteriophage-treated wounds showed a significant reduction in bacterial load compared to the control groups. There was a marked improvement in the wound healing process in the phage-treated group, evidenced by faster wound closure and better tissue regeneration.
Burn wound	*P. aeruginosa *and *K. pneumoniae*	PA phagocytic monomer cocktails Single bacteriophage (kpn 1, kpn 2, kpn 3, kpn 4 and kpn 5)[87]	Both the single phage and the phage cocktail significantly reduced the bacterial load in the burn wounds compared to the control group, faster wound healing and better tissue regeneration.
Tumor	*Fusobacterium nucleatum* (promotes chemotherapy failure)	Bacteriophage-guided biotic-abiotic hybrid nanomaterials [88]	The treatment with the bioinorganic hybrid bacteriophage resulted in a significant reduction in tumor growth in mouse models of colorectal cancer.
Bacterial infections	P. aeruginosa and K. pneumoniae	Phage LysGH15 [89]	The ointment showed significant antibacterial activity against MRSA, effectively reducing bacterial counts in infected skin wounds.
Lung infection, cystic fibrosis	P. aeruginosa	Bacteriophages φMR299-2 and φNH-4 [90] Gold nanoparticles (AuNPs) [91]	Both ϕMR299-2 and ϕNH4 significantly reduced the bacterial load in the lungs of infected mice. The gold nanoparticles exhibited significant anti-biofilm activity against the tested bacterial pathogens.

Challenges and limitations

A particular phage can only be effective against a limited range of bacteria due to the high strain specificity exhibited by bacteriophage. Therapy cannot start until infecting bacteria have been identified. Similar to how they can get resistant to antibiotics, bacteria can develop resistance to phages. PT may become less effective over time as bacteria evolve to avoid phage infection. Genetically engineered phages, synthetic and phage cocktails need to be studied more, as little is known about altered synthetics combined with natural organisms. One more limitation is that chronic wound infections with fungal co-infection cannot be treated with PT. This necessitates ongoing research and development of new phages or phage cocktails to combat emerging bacterial resistance. Producing phages on a large scale and ensuring their quality and purity can be challenging. Ensuring consistency in treatment outcomes requires the standardization of phage preparations for clinical use. Phage selection and specificity, phage resistance, regulation hurdles, production, and standardization are major challenges in PT. Personalized approaches are often necessary, but they can be more costly and complex [92].

Future perspectives

Customized PT can be enabled by advances in genome sequencing and analytics by focussing on particular bacterial strains that cause infections and tailoring treatment based on the patient’s microbiome and infection profile. By utilizing their complementary mechanisms, PT, in conjunction with antibiotics, can increase treatment efficiency and decrease bacterial resistance, providing a more comprehensive and effective treatment choice. Genetic engineering and synthetic biology techniques enable phage alterations for better therapeutic properties, focussing on bacterial resistance, improving pharmacokinetics and transport efficiency, and expanding the therapeutic potential of PT. Research in this field is mainly concentrated on developing techniques to penetrate and disrupt bacterial biofilm; these processes have the potential to overcome antibiotic resistance mediated by biofilm and improve therapeutic outcomes.

As more clinical data show the safety and efficacy of PT, interest in phage-based intervention approval and clinical translations is growing. Standardized methods for phage production and quality control must be established, regulatory pathways must be streamlined, and well-designed clinical trials must be conducted before PT may become a widely used antimicrobial treatment option.

## Conclusions

Wound infections are difficult to manage due to the presence of biofilm, poor penetration of antibiotics, and MDR bacteria. Pre-clinical and clinical trials expanded the therapeutic efficiency of phage therapy in chronic wounds. However, there is a need to carry out these trials extensively. Only then can we arrive at a proper treatment plan for these wounds? As phages are host-specific, easy to manipulate, and safe for human therapy, they are ideal tools for eradicating multidrug-resistant microbes. The development of standardized protocols for phage production and selection processes, the evaluation of the safety and effectiveness of clinical trials, and the incorporation of PT into the current chronic wound treatment framework are the major translational aspects. Regulatory agencies and healthcare providers must work together to ensure patient safety, effectiveness, and accessibility for patients with chronic wound infections. This translational potential supports PT as an alternative to antibiotics for the management of complex wound infections. Successful phage therapy depends on the selection of phage, reliable phage delivery at the site of infection, taking care of the duration of therapy, ideal dose, sufficient titers, and interactions with antibiotics while following the proper regulations.
